# How does Community-Led Total Sanitation (CLTS) promote latrine construction, and can it be improved? A cluster-randomized controlled trial in Ghana

**DOI:** 10.1016/j.socscimed.2019.112705

**Published:** 2020-01

**Authors:** Miriam Harter, Jennifer Inauen, Hans-Joachim Mosler

**Affiliations:** a Eawag, Swiss Federal Institute of Aquatic Science and Technology, Ueberlandstrasse 133, 8600, Dübendorf, Switzerland; b Department of Psychology, University of Bern, Fabrikstrasse 8, 3012, Bern, Switzerland

**Keywords:** CLTS, RANAS model, Behaviour change, Latrine ownership, Psychosocial determinants, Multilevel mediation analysis, Ghana

## Abstract

**Rationale:**

Open defecation is connected to poor health and child mortality, but billions of people still do not have access to safe sanitation facilities. Community-Led Total Sanitation (CLTS) promotes latrine construction to eradicate open defecation. However, the mechanisms by which CLTS works and how they can be improved remain unknown. The present study is the first to investigate the psychosocial determinants of CLTS in a longitudinal design. Furthermore, we tested whether CLTS can be made more effective by theory- and evidence-based interventions using the risks, attitudes, norms, abilities, and selfregulation (RANAS) model.

**Methods:**

A cluster-randomized controlled trial of 3216 households was implemented in rural Ghana. Communities were randomly assigned to classic CLTS, one of three RANAS-based interventions, or to the control arm. Prepost surveys at 6-month follow-up included standardized interviews assessing psychosocial determinants from the RANAS model. Regression analyses and multilevel mediation models were computed to test intervention effects and mechanisms of CLTS.

**Results:**

Latrine coverage increased pre-post by 67.6% in all intervention arms and by 7.9% in the control arm (p < .001). The combination with RANAS-based interventions showed non-significantly greater effects than CLTS alone. The effects of CLTS on latrine construction were significantly mediated by changes in four determinants: others' behaviour and approval, self-efficacy, action planning and commitment. Changes in vulnerability, severity, and barrier planning were positively connected to latrine construction but not affected by CLTS.

**Conclusion:**

This study corroborates the effectiveness of CLTS in increasing latrine coverage, and additional activities can be improved further. Behaviour change techniques within CLTS that strengthened the relevant factors should be maintained. The study also recommends interventions based on the RANAS approach to improve CLTS. Further research is needed to understand the effects of RANAS-based interventions combined with CLTS at longer follow-up

## Highlights

• Randomized-controlled trial examined Community-Led Total Sanitation (CLTS) in Ghana.
• CLTS increased latrine coverage by 67.6%.
• CLTS achieved changes in psychosocial determinants based on a psychosocial model.
• Changes in psychosocial determinants explain success of CLTS.
• Changes in factors such as social norms explain why participants construct latrines.

## 1 Introduction

The global community has set itself the goal of providing access to safe sanitation facilities for all by 2030 (Goal number 6, Sustainable Development Goals). The updated status report on the Sustainable Development Goals of 2017 acknowledged that this goal will not be reached (WHO & UNICEF, 2017). In 2015, 2.3 billion people lacked access to basic sanitation services, and 892 million people practised open defecation (WHO & UNICEF, 2017). As a result of open defecation, 1.8 billion people worldwide use drinking water that is contaminated with faecal bacteria (WHO & UNICEF, 2017). Every year, the deaths of approximately 361,000 children under five could be prevented by safe sanitation (Pr ü ss-Ust ün et al., 2014). Wolf et al. (2018) show that among adults, sanitation interventions reach up to 25% reduction in diarrheal diseases, and evidence is presented that the effect is higher in communities with higher latrine coverages. This fact shows that open defecation is not only an individual health hazard. Individuals might change from open defecation to latrine usage, but as long as their neighbours continue defecating in the latrine, users remain threatened by faecal contamination of water bodies and food (Coffey et al., 2017; Julian, 2016; Root, 2001). Therefore, collective behaviour change is required to achieve an environment free of open defecation.

Community-Led Total Sanitation (CLTS) is a participatory approach that evokes collective behaviour change in rural settings. Originally developed in Bangladesh in 2008 (Kar and Chambers, 2008), it has since been adopted globally (USAID, 2018). The approach combines a range of activities that are implemented by local facilitators at a community level in three phases (Kar and Chambers, 2008). In the initial phase, pre-triggering, each community is visited, and information is gathered about the population and their readiness for behaviour change. In the second phase, triggering, this information is used to adjust participatory behaviour change techniques (BCTs). These techniques are then applied during a community event such as community mapping or a transect walk along which the community is confronted with faecal contamination. The optimal outcome of this community meeting, also called the triggering event, is an increase in community members’ awareness that “they are eating their own faeces” ((Kar and Chambers, 2008), page 35). This realization should lead to a change in sanitation conditions by constructing latrines (Kar and Chambers, 2008). Third, during the post-triggering phase, facilitators support the community in achieving the status of an “open defecation free community,” by helping in the construction of latrines. The original CLTS process works without any subsidies (Kar, 2003).

Given the wide adoption of CLTS (Cavill, 2018), its effectiveness has still rarely been scientifically investigated. The few rigorous scientific studies of CLTS's effectiveness have produced diverse and ambiguous findings (Pickering et al., 2015; USAID, 2018; Venkataramanan et al., 2018). A recent meta-analysis on the impact of sanitation campaigns showed that CLTS typically increases latrine coverage by 6–12% and can reach up to 30% (Garn et al., 2016). Another recent review shows that most of the literature on CLTS is grey literature and that only 7% can be categorized as scientific studies (Venkataramanan et al., 2018). This review concludes that CLTS still lacks a systematic and detailed understanding of the mechanisms of behaviour change, which is common practice according to the National Institutes of Health (NIH); the NIH instead recommends to investigate mechanisms of behaviour change strategies (Nielsen et al., 2017). Like any other behaviour change campaign, CLTS seeks to change people's mindsets. It aims at to evoke the need to construct and use latrines to achieve a healthy, faeces-free environment. Nevertheless, what is yet unknown is which elements of CLTS convince people to change and what changes in people's mindsets actually prompt them to construct latrines.

One theoretical framework that explains such changes in mindsets in the sector of water sanitation and hygiene (WASH) is the risks, attitudes, norms, abilities, and self-regulation (RANAS) model of behaviour change (Mosler, 2012; Mosler and Contzen, 2016). It combines existing theoretical models of behaviour change, such as the health action process approach (Schwarzer, 2008) and the theory of planned behaviour (Ajzen, 1991). The core concept of the RANAS model is that behaviour change is driven by various psychosocial determinants that need to be in favour of a new behaviour (Mosler, 2012). These determinants are clustered in five factor blocks: 1) Risk factors include individuals' health knowledge, its perceived severity, and their vulnerability to it; 2) attitude factors include feelings about the new behaviour and the perceived costs and benefits of performing it; 3) norm factors include people's perceptions of others' behaviour and their perceived (dis)approval when an individual shows the new behaviour; 4) the ability factor block includes the knowledge of how to perform the behaviour and confidence in starting a behaviour, continuously performing it, and recovering it after relapse; and 5) the self-regulation factor block contains the individual's action plans for the behaviour, how he or she deals with barriers, self-monitoring (e.g., action control) and remembering the new behaviour and the commitment to performing the behaviour. These psychosocial factors are used to develop theory- and evidence-based behaviour change interventions. Differences are observed between the psychosocial determinants of people who already show the new behaviour and those who do not yet show it. The determinants that show the greatest differences are those targeted in behaviour change campaigns. The RANAS model offers a catalogue of BCTs that are linked to corresponding psychosocial determinants. Those techniques can be combined to create complex data-driven and population-tailored interventions. Campaigns planned following the RANAS model have demonstrated success in changing behaviour and evoking new and sustainable habits in various contexts of the WASH sector (Contzen et al., 2015; Friedrich et al., 2018; Inauen and Mosler, 2014; Lilje and Mosler, 2018). The combination of such data-driven and population-tailored interventions with the CLTS approach might be even more powerful and efficient in changing behaviour than CLTS alone. Such combinations might provide deeper insights into the mechanisms by which CLTS evokes change and might lead to further improvements.

This study therefore investigates the effectiveness of CLTS and combines CLTS with data-driven and population-tailored interventions following the RANAS approach. We hypothesize that CLTS in any combination motivates people to construct latrines (H1) and that the combination of CLTS plus RANAS-based interventions are more effective in evoking latrine construction than CLTS alone (H2). We test these hypotheses by comparing the effects of four interventions with a control arm undergoing no intervention. In addition, this study is the first to investigate how CLTS promotes latrine construction by examining which psychosocial mechanisms of the RANAS model explain its effectiveness.

## 2 Method

### 2.1 Study design

A cluster-randomized controlled trial was conducted with two panel surveys that measured intervention effects pre-post. The trial comprised four intervention arms and one control arm: (1) classic CLTS, (2) CLTS combined with an extended public commitment, (3) CLTS combined with a household action planning, (4) CLTS combined with public commitment and household action planning, and (5) a control arm with no intervention. The baseline survey was conducted in February and March 2016, and the interventions were implemented between July and November 2016. Classis CLTS was implemented first, and the three RANAS-based intervention arms were implemented after completion of the classic CLTS. A first follow-up survey was conducted in February to March 2017, between four and six months after the implementation. Long-term effects were measured with a second follow-up survey 14–16 months after implementation, in February to March 2018, but those results are not included in this article's analysis. Participants were allocated randomly to intervention arms on a regionally clustered basis to avoid spill over effects between participants of different interventions. This trial was approved by the Ethical Review Committee of the Ghana Health Service (GHS-ERC: 05/01/2016) and the Ethical Board of the University of Zurich, Switzerland.

### 2.2 Participants

This trial was conducted in two districts of the Northern Region of Ghana, Sawla-Tuna-Kalba, and Bole. The two districts were selected by the implementing NGO because CLTS had not been previously implemented in either. Within the two districts, the regional government selected 132 communities based on the following criteria. Communities needed a) to be accessible by car or motorbike from the two district capitals to render the trial practicable and b) to have at least 25 households (the minimum cluster size). Within the communities, household selection followed the random route method (Hoffmeyer-Zlotnik, 2003). Data collectors selected every third household on their way starting from the centre of the community, and each data collector headed in a different direction. Respondents needed to be over 18 years and resident in the community for at least three months to be able to answer questions concerning community characteristics. Male and female respondents were considered equally because the decision to construct latrines might be taken by both. If no one was at home or the person present refused to participate, the next household was selected. Written informed consent was obtained from all participants.

### 2.3 Sample size

**Fig. 1:**
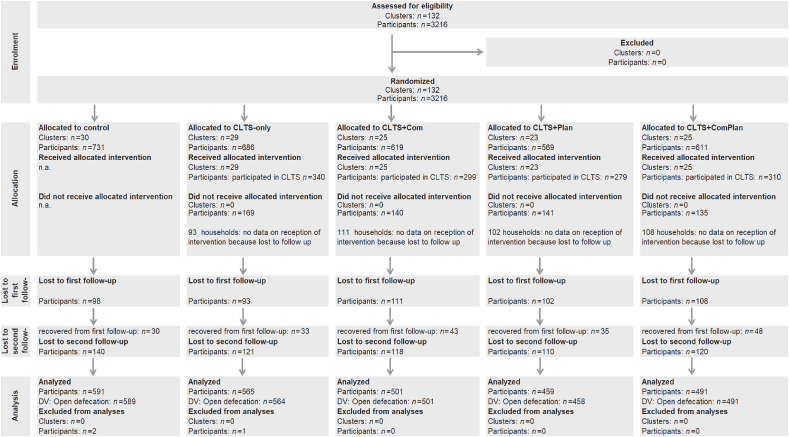
Flow diagram of the sample, *Note:* DV = Dependent variable. Lost participants at follow-up (*n =* 512) were compared to the remaining sample (for drop-out analysis see Table S5 in supplementary material). No clusters were lost to follow-up.

We calculated our sample size a priori for a multilevel longitudinal model with repeated measurements and a cluster-randomized controlled trial design for the primary dichotomous outcome variable, latrine ownership (Spybrook et al., 2011). We used Optimal Design Plus Version 3.0 to calculate the sample size. Based on the literature (USAID, 2018), we assumed a medium intervention effect of 20% (Cohen's *d* = 0.63) and a drop-out rate of 20%. Assuming power of 80%, a significance level of 5% (two-tailed), and an intra-cluster correlation of ρ = 0.20, there was a sample size of 3125 households—25 clusters for each of the five intervention arms, with 25 households per cluster. The actual sample size exceeds the calculated sample size by 91 cases, which resulted from practical decisions during the field survey. We decided to include more communities because during the survey, we found that some communities did not include 25 households as expected. To obtain the sample of 3125 households required by our statistical approach, seven additional communities were included, and 25 households interviewed in each, if possible. The total and final sample size was therefore 3216 households. Intra-class correlations are displayed in Table S3 in the supplementary material. The flowchart in Fig. 1 shows sample development at cluster and individual levels.

### 2.4 Interventions

The RANAS approach was used to develop interventions based on the data gathered about the target population at the baseline survey. The RANAS approach identifies the psychosocial determinants that steer latrine construction and then selects BCTs that target these determinants (Mosler and Contzen, 2016). Baseline data revealed that latrine construction was steered by determinants of the social and physical context, of the risk factor block, and of attitudes, norms, and self-regulation factor blocks. The determinants identified and the corresponding BCTs based on Michie et al. (2013) and Abraham and Kools (2011) can be assessed in the supplementary material in Table S1. BCTs were combined in two interventions and added to the classic CLTS intervention, which resulted in five intervention arms: classic CLTS (CLTS-only), three CLTS arms with additional RANAS-based interventions, and a control arm (no intervention). The RANAS-based interventions added to CLTS were an extended public commitment (CLTS + RANAS-Com), household action planning for latrine construction (CLTS + RANAS-Plan), and the combination of both (CLTS + RANAS-ComPlan). All the intervention protocols were discussed and agreed between the study manager and Global Communities Ghana, the local partnering NGO. Global Communities trained facilitators and coordinated implementation of all the intervention arms with the support of the first author for the RANAS-based interventions. The RANAS-based interventions were first piloted in 12 communities and implemented at scale after final revision of the intervention materials. Intervention protocols can be found online (https://osf.io/gdcqs/?view_only=eb1238cbaebf403c8618f971e500c206).

#### 2.4.1 Community-Led Total Sanitation

All three phases of CLTS were implemented according to the CLTS manual (Kar and Chambers, 2008). During the pre-triggering phase, data about the community was collected by the facilitators, such as population size and numbers of existing latrines. Subsequently, all members of the community were invited to a triggering event. The facilitators mainly used three activities for the triggering event. Firstly, an improvised map was drawn on the ground, and community members located their houses on it, and then added places they used for open defecation. Secondly, medical costs were calculated for diarrheal diseases and compared to costs for latrines built from local materials. Finally, a community action plan was established, which defined a date by which the community wanted to achieve the status of an open defecation free community. Individuals that showed leadership qualities were selected as natural leaders and trained by Global Communities to better support latrine construction in their communities. In the post-triggering phase, facilitators visited the community each week to support and train community members and natural leaders on latrine construction, and to help solve challenges that community members faced during this process. CLTS formed part of all four intervention arms, and public commitment and planning were added to this procedure after the triggering event.

#### 2.4.2 Public commitment

Public commitment (CLTS + RANAS-Com) involved participants stepping up in front of the community after the triggering event and showing their commitment to construct latrines. The facilitators were advised to praise the first volunteers as progressive and respected. The remaining community members clapped for those who committed publicly to constructing latrines. The commitment to construct a latrine was made visible by providing stickers to those who had promised to do so. The sticker was to be located where it would be visible to by-passers. After the latrine was constructed, owners received a white flag from the facilitators, which was hung on the latrine.

#### 2.4.3 Household action planning

The facilitators worked in teams of two and visited every household in the communities allocated to this intervention arm in the week after the triggering event (CLTS + RANAS-Plan). During their visits, a detailed household action plan was developed with the person responsible for latrine construction in each household. The facilitator supported the household member in choosing a latrine type, estimating the time needed for each step of construction, and considering which materials would be needed and who would be responsible of each step of construction. Both facilitators and household members signed the action plan. It also served as a monitoring tool for both facilitators and household members by which the progress of latrine construction was recorded. The plan was copied for the facilitator, and one plan remained with the household.

#### 2.4.4 Combination of public commitment and household action planning

The two procedures explained above, the public commitment and the household action planning, were combined in the fourth intervention arm (CLTS + RANAS-ComPlan). After the triggering event including public commitment, the facilitators returned to the community the following week and completed the household action planning as described above.

#### 2.4.5 Control group

Communities that were assigned to the control group did not receive any intervention. After completion of the long-term follow-up survey, all control communities received classic CLTS.

### 2.5 Data collection and study measures

For both baseline and follow-up surveys, the research manager conducted one week of training with 33 local data collectors. The training included questionnaire discussion and translation into interview languages (Brefo, Dagaare, Gonja, Waale, Safalba, and Twi), role plays on interview techniques, and discussion of ethics. The training also included two-days pre-testing of the instruments under local conditions. A total of seven teams each with three to five data collectors worked in the two districts, and every team was accompanied by one supervisor (research manager, local field coordinators, interns, and master's students). Interviews were structured, conducted face to face, and lasted 50 min on average. The following outcome measures were assessed at baseline and follow-up: behaviour (latrine construction, usage, and open defection frequencies), information on the social context, and RANAS psychosocial determinants. The surveys also included short observations of the hygiene situation and the household latrine, where applicable.

#### 2.5.1 Latrine construction

The question *Does your household have its own latrine?* served as an indicator for a constructed household latrine at first follow-up. Latrine construction was coded with 0 = no household latrine and 1 = household latrine (completed or still under construction). This self-reported statement was verified by observations of the data collectors (accordance rate of 93.6%).

#### 2.5.2 Psychosocial determinants

Psychosocial determinants were assessed using the RANAS approach (Mosler and Contzen, 2016). All items were answered on 5-point Likert scales. We used a visual scale with five black spots of varying sizes to help respondents choose one of the answering options. Every answer option was read out to the respondent and indicated on the visual scale (scale in supplementary material, Fig. S1). Sample items for each factor are displayed in Table 1; for a complete picture of all items used for this analysis, please consult Table S2 in supplementary material. Psychosocial determinants were assessed with one or two and more items, which were combined to scales by averaging item scores (Cronbach's Alpha for the combined items are reported in the supplementary material).

**Table 1:** Sample items for psychosocial determinants based on the RANAS-model of behaviour change.

Risk factor block	
Vulnerability	Generally, how high do you think is the chance that you get diarrhoea? *1 = not at all high to 5 = very high*
Severity	Imagine that you have diarrhoea, how severe would be the impact on your life? *1 = not at all severe to 5 = very severe*
Health knowledge	Could you please tell me for each of the following aspects whether it is a cause of diarrhoea or not (e.g., water contaminated by bacteria)? *1 = Yes; 2 = No; 99 = I don't know*
Attitudes factor block	
Feelings	How proud are you of your own latrine? *1 = not at all proud to 5 = very proud*
Beliefs about costs and benefits	How expensive is it to construct your own latrine? *1 = not at all expensive to 5 = very expensive*
Norm factor block	
Other's behaviour	How many of your relatives within your community constructed an own latrine? *1* = *(almost*) *nobody to 5* = *(almost*) *all*
Other's approval	How much do people who are important to you (e.g. family, parents, friends) approve that you construct a latrine? *1 = approve not at all to 5 = approve very much*
Abilities factor block	
How-to-do-knowledge	Which of the following features are necessary for a hygienic latrine (e.g., vent pipe)? *1 = Yes; 2 = No; 99 = I don't know*
Confidence in performance	How confident are you that you can construct a latrine even if this is difficult (e.g. gathering the materials)? *1 = not at all confident to 5 = very confident*
Confidence in continuation	How confident are you that you can finish the construction of a latrine even if problems arise (e.g. you run out of money)? *1 = not at all confident to 5 = very confident*
Confidence in recovering	Imagine that the latrine got damaged. How confident are you that you will be able to repair the latrine again? *1 = not at all confident to 5 = very confident*
Self-Regulation factor block	
Commitment	How committed are you to constructing your own latrine? *1 = not at all committed to 5 = very committed*
Action Planning	Do you have a plan how you will gather the materials for the latrine construction? *1 = Yes;2 = No*
Barrier Planning	Do you have a plan how you can construct a latrine if you are running out of materials? *1 = Yes;2 = No*

*Note.* The RANAS model also includes Remembering and Action control within the Self-regulation block. Neither psychosocial determinant was found to be appropriate for latrine construction, so neither was assessed.

### 2.6 Data analyses

To test intervention effects on latrine construction at follow-up, a generalized linear mixed model with a binomial distribution was fitted using IBM SPSS Statistics 24 (Rabe-Hesketh et al., 2004). This model accounted for the nested nature of our data (households within communities) and allowed modelling the heterogeneity between communities. To test whether CLTS was more effective than the control group (H1), a dummy-coded independent variable was entered that was coded 1 for the control arm and 0 for the CLTS arms. Three additional dummy variables tested whether the CLTS + RANAS intervention arms were more effective than CLTS alone (H2). They were coded 1 for the intervention arm and 0 for the CLTS-only arm (for the coding of intervention arms see Table S3 in supplementary material). Latrine construction was the outcome variable (0 = no own household latrine, 1 = own household latrine [finished or under construction]). Random effects were included, using a variance components correlation structure. The syntax for the model calculation can be provided upon request. Missing values were found to be completely missing at random and imputed using the multiple imputation approach (results are displayed in Table S6 in supplementary material).

To identify which psychosocial determinants of the RANAS model were changed by CLTS and how this change resulted in latrine construction, multilevel mediation analyses were fitted using Mplus Software (Version 8), which uses a full-information-maximum likelihood method by default (Asparouhov and Muthén, 2010; Larsen, 2011). Mediation analysis tests the causal mechanisms of an intervention on the outcome (here, latrine construction) by partitioning the effect in direct and indirect effects via a mediator—in this case, the psychosocial determinants (Hayes, 2013). It investigates whether the intervention effect diminishes when adjusted for a mediating variable. The hierarchical nature of our data prompted us to use a 2-1-1 multilevel structural equation model in which the independent variable, the intervention, varied at level 2 (community), and the mediators (changes in psychosocial determinants) and outcome (latrine construction) varied at level 1 (household) (Preacher et al., 2010). By definition, the effect of the intervention on the mediators occurs at the intermediate level (X-M). Conversely, the effect of the mediators on the outcome (M-Y) can additionally occur at level 1 (households). Thus, the M-Y relationship was allowed to vary across different communities and individuals. Confidence intervals (*CI*) for the indirect effects were calculated using the Monte Carlo Method (Koo and Li, 2016). The CLTS intervention was coded 0 = control arm vs. 1 = all four arms with CLTS. To reflect the changes achieved by the interventions on psychosocial factors, baseline values were deducted from follow-up values. Accordingly, all pre-post differences in psychosocial factors within households ranged between −1 and 1, so that positive values reflect increases and negative values decreases in the psychosocial determinants. All level 1 determinants were grand-mean centred (Preacher et al., 2010). To test for multicollinearity, we estimated the variance inflation factors that were <2 for all determinants, meaning that multicollinearity was not a problem for these data (correlation matrix and variance inflation factors values are displayed in the supplementary material in Table S3). Analysis of intra-class correlations revealed high variance within communities (all values < 0.5) (Koo and Li, 2016).

## 3 Results

### 3.1 Sample characteristics

The sample comprised 42% female respondents, aged *M* = 44.6 years (*SD* = 14.3); 20.8% were literate. The average household size was 8.9 members (*SD* = 5.5). Some 49.2% reported Christianity as their religion, 22% Islam, and 16.2% traditional religions. Most of the respondents were farmers (80.4%), with an average monthly income of 224 GHS per household (*SD* = 1020; equivalent of USD = 50). Baseline characteristics of individuals of intervention and control arms are displayed in Table 2. The five arms differed in all characteristics except age. Effect sizes for these differences were small (Cohen, 1992). Nevertheless, sensitivity analyses were conducted for the main effects analysis, and the mediation models adjusting for these covariates. The results showed that including the baseline characteristics did not substantively change the findings.

**Table 2:** Socio-demographic characteristics in intervention and control arms (*n* = 3216).

Characteristic	Control arm	CLTS-only (*n =* 686)	CLTS + RANAS-Com (*n =* 619)	CLTS + RANAS-Plan (*n =* 569)	CLTS + RANAS-ComPlan (*n =* 611)	Χ^2^	*p*	*d*
Occupation						123.8	<.001	.40
Farming	66%	86%	83%	83%	84%			
Other (trading, mining, fishing)	34%	13%	16%	16%	16%			
Religion						102.2	<.001	.36
Islam	39%	25%	18%	20%	22%			
Christian	43%	51%	53%	50%	48%			
Traditional	13%	15%	22%	23%	23%			
Atheist	3%	6%	5%	6%	5%			
Female	49%	42%	37%	37%	44%	28.5	<.001	.12
Literacy	25%	22%	19%	21%	16%	17.1	.002	.12
	*M* (*SD*)	*M* (*SD*)	*M* (*SD*)	*M* (*SD*)	*M* (*SD*)	*F*	*p*	*d*
Age	44.4 (16.3)	43.8 (15.7)	45.3 (15.8)	43.8 (15.9)	45.6 (17.1)	1.1	.37	.01
Income	268.5 (527.3)	182.7 (311.1)	182.2 (290.3)	197.3 (389.7)	170.3 (296.3)	7.6	<.001	.22
Household size	8.4 (4.6)	8.6 (4.6)	9.2 (5.1)	8.9 (4.9)	8.5 (5.0)	2.8	.020	.08

*Note:* Effect sizes for independent means according to Cohen (1992): *d =* 0*.*2 (small), *d=.5* (medium), *d =* 0*.*8 (large). For sensitivity analysis, all determinants were included.

### 3.2 Intervention exposure

Intervention protocols showed that overall 67.8% (*n* = 1228) of the respondents participated in the CLTS triggering events (see also intervention flow diagram in Fig. 1). For the Action plans, reports showed that of the respondents that were assigned to this intervention, 41.5% (*n* = 401) received a household action plan. Of those respondents assigned to the Public commitment, 42.8% (*n =* 431) received a sticker, and 12.6% (*n =* 127) received a flag.

### 3.3 Intervention effects

**Fig. 2:**
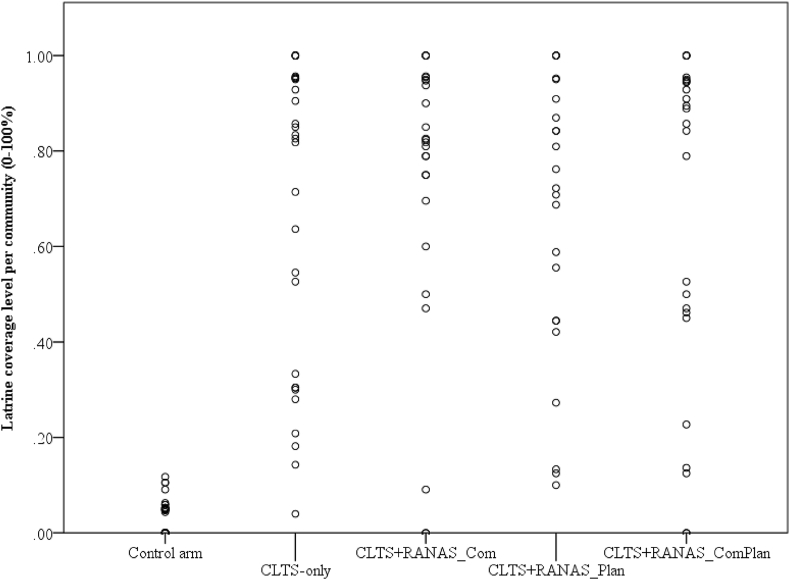
Latrine construction variability per community and intervention arm. *Note.* Each dot resembles one community.

Intervention effects are displayed in Table 3. In the CLTS arms, *M* = 68.2% (*SD* = 30.8%) had constructed a latrine at follow-up (baseline latrine coverage *M =* 3.2%, *SD =* 0.2%). Latrine construction in the control arm was significantly lower (*M* = 7.9%, *SD* = 8.1%). The three CLTS + RANAS intervention arms exhibited a similar proportion of household latrines as CLTS alone (*M =* 65.5%, *SD =* 31.5%) and did not differ significantly: CLTS + RANAS-Com (*M =* 73.2%, *SD =* 29.0%, *p =* .597), CLTS + RANAS-Plan (*M =* 67.1%, *SD* = 27.8%, *p =* .964) and CLTS + RANAS-ComPlan (*M =* 67.7%, *SD =* 33.5%, *p =* .962). The random effects indicated that the level of community latrine construction varied significantly between communities (*Estimate* = 2.76, *SE* = 0.44, *p* < .001*, 95% CI* (2.02, 3.77). For example, in the intervention arm CLTS + RANAS_Com, the range of latrine coverage in those intervention communities ranged from 0 to 100%. Fig. 2 graphically portrays this variance.

**Table 3:** Parameter estimates for multilevel model of intervention effects on latrine ownership at follow-up.

Fixed Effects (intercept, slopes)	B (*SE*)	*p*	OR	95% CI for OR	
				LL	UL
Intercept: CLTS effect only^a^	2.54 (1.26)	0.044	12.62	1.07	148.71
Effect of control arm compared to CLTS only^b^	−3.83 (0.42)	<0.001	0.02	<0.01	0.05
CLTS + RANAS-Com compared to CLTS only^c^	0.27 (0.52)	0.597	1.31	0.48	3.60
CLTS + RANAS-Plan compared to CLTS only^d^	−0.02 (0.49)	0.964	0.98	0.38	2.54
CLTS + RANAS-ComPlan compared to CLTS only^e^	0.03 (0.55)	0.962	1.03	0.35	3.00
			95% CI		
	Estimate (*SE*)	*p*	LL	UL	
Random intercept^f^	2.76 (0.44)	0.000	2.02	3.77	
Residual variance^g^	1 (.)	.	.	.	

*Note. N =* 2,703, *B* = unstandardized regression coefficients. *CI* = Confidence interval. *OR* = Odds ratio. Probability distribution: binomial, link function: logit. All *p-*values are two-tailed. Outcome variable: Latrine construction 0 = no latrine, 1 = latrine (finished or under construction).

a Intercept: Probability for latrine construction at follow-up when CLTS was received.

b CLTS: 0 = CLTS arms, 1 = control arm.

c CLTS + RANAS-Com: 0 = other arms, 1 = CLTS plus RANAS-based public commitment.

d CLTS + RANAS-Plan: 0 = other arms, 1 = CLTS plus RANAS-based household action planning.

e CLTS + RANAS-ComPlan: 0 = other arms, 1 = CLTS plus RANAS-based public commitment + household action planning.

f Random intercept: variation in latrine construction between communities.

g Residual variance: variation in latrine construction between individuals per definition 1 (binomial distribution).

### 3.4 Explaining effects of CLTS on latrine construction through changes in RANAS-based psychosocial determinants

**Fig. 3:**
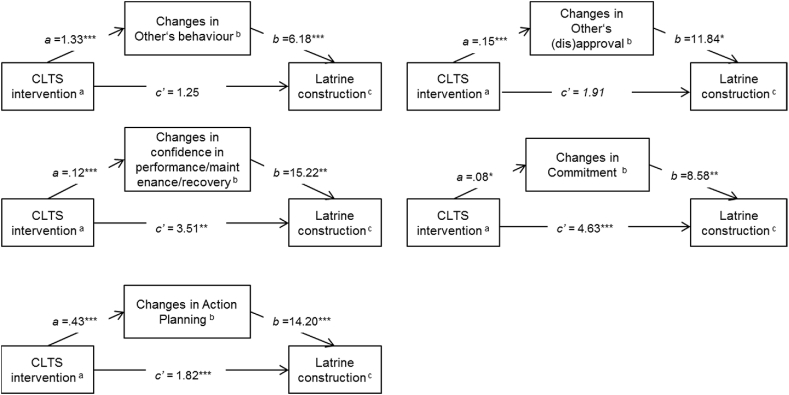
Single-mediation models of the intervention effect on latrine construction mediated by changes on RANAS-based psychosocial determinants, ^a^ CLTS intervention 0 = control arm, 1 = all interventions with CLTS ^b^ Changes on the mediator (follow up-baseline), range −1 to 1, ^c^ Latrine construction was coded 0 = no latrine, 1 = latrine (finished or under construction), Significance levels: *p < .05, **p < .01, ***p < .001.

The main effects analysis showed no differences between the CLTS arms in latrine construction. Previous analysis additionally indicated that the changes on psychosocial determinants were not distinguishable between different arms (see Table S8 in supplementary material). Consequently, the CLTS intervention arms were combined and compared to the control group. Changes in the values for five of the psychosocial determinants of the RANAS model mediated the effects of CLTS on community latrine construction (see Fig. 3 and Table S7 in supplementary material). The intervention significantly increased community-level perceptions that others owned a household latrine (others' behaviour, *Β* [*SE*] *=* 0.28 [0.05], *p <* .001) and increased the perception that community leaders approved latrine construction (others' approval, *Β* [*SE*] = 0*.*15 [0.04], *p <* .001). The intervention also increased confidence in constructing, maintaining, and repairing a latrine (*Β* [*SE*] *=* 0*.*12 [0.02]*, p <* .001) and strengthened people's commitment to constructing their own household latrines (*Β =* 0.08 [0.04], *p = .*041). Finally, the intervention promoted the formation of action plans for latrine construction (*Β* [*SE*] *=* 0.43 [0.09], *p <* .001). These changes individually increased the probability of communities having higher latrine coverage (others' behaviour: *Β* [*SE*] *=* 6.18 [0.45]*, p* < .001; others' approval: *Β* [*SE*] *=* 11.84 [4.76]*, p* = .013; confidence in performance/maintenance/recovery: *Β* [*SE*] *=* 15.22 [4.45], *p =* .001; commitment: *Β* [*SE*] *=* 8.58 [3.12], *p =* .006; action planning: *Β* [*SE*] *=* 14.20 [0.34]*, p <* .001). Indirect effects were found to be significant for others' behaviour *(Β* [*SE*] = 1.75 [0.34], 95% *CI* = 1.08, 2.42), confidence in construction/maintenance/recovery *(Β* [*SE*] = 1.75 [0.69], 95% *CI* = 0.39, 3.10), commitment *(Β* [*SE*] = 0.67 [*0.42*], 95% *CI* = −0.18, 1.52), and action planning *(Β* [*SE*] = 6.03 [1.31], 95% *CI* = 3.47, 8.59). Changes in these determinants mediated the effect of CLTS on latrine ownership. The relationship between changes in the psychosocial determinants and the probability of latrine coverage was significantly different within communities for all mediators considered for analysis (estimates for random effects on level 1 are displayed in Table S7 in the supplementary material). The intervention further had a significant effect on Feelings (*Β* [*SE*] *=* -0.05 [0.01]*, p <* .001), so that CLTS participants experienced a loss in their belief that a latrine would make them more respected by other community members and that owning a latrine would make them feel proud. But, this change was not associated with the probability of latrine construction (*Β* [*SE*] = -21.20 [11.91]*, p* = .075). The other determinants were not significantly affected by the CLTS intervention. Changes in some of the behavioural determinants were found to be relevant to the probability of constructing a household latrine but were not significantly addressed by the CLTS intervention. People with higher negative changes in Vulnerability were more likely to construct latrines (*Β* [*SE*] = -6.43 [1.73], *p <* .001). People who had stronger positive changes in their perception of the severity of getting diarrhoea were more likely to construct latrines (*Β* [*SE*] = 6.36 [2.79]*, p =* .023) as were people with higher increases in their felt abilities to cope with problems arising during latrine construction (*Β* [*SE*] *=* 7.64 [3.34], *p =* .022).

## 4 Discussion

This study investigated the intervention effect of a classic CLTS intervention on latrine construction. It is the first randomized controlled trial to examine the effects of population-tailored and data-driven behaviour change techniques in addition to classic CLTS. It is also the first study to investigate how CLTS promotes latrine construction by changes in psychosocial determinants. The results showed once more that CLTS is powerful in evoking latrine construction, as reported by previous studies (Bongartz et al., 2016; Crocker et al., 2016; Pickering et al., 2015; USAID, 2018; Venkataramanan et al., 2018). The 67% latrine coverage in the intervention communities, compared to just 7.9% in control communities, is higher than in other CLTS interventions in Ghana (45%, (USAID, 2018)) or to other sanitation campaigns (30%, (Garn et al., 2016)). It is comparable to CLTS outcomes in other countries, such as Mali (65%) or Malawi (100% (USAID, 2018)). Yet, our second hypothesis could not be confirmed: Additional campaign activities based on the RANAS approach for systematic behaviour change (Mosler, 2012; Mosler and Contzen, 2016) tended to show increased latrine coverage but was not significant compared to CLTS alone (see Fig. 2 and Table 3). This finding is surprising considering previous studies that found highly significant RANAS-based intervention effects in various cultural settings and behavioural contexts—for example, on collecting arsenic-free drinking water in Bangladesh (Inauen and Mosler, 2014), hand-washing interventions in Burundi (Sonego and Mosler, 2014), shared latrine cleaning in Uganda (Tumwebaze and Mosler, 2015), and disinfecting drinking water in rural Chad (Lilje and Mosler, 2018). One reason that no additional effects were observed for the RANAS interventions is low intervention fidelity. As the implementation protocols of the implementing NGO indicate (see also flow diagram in Fig. 1 and intervention exposure), for example, only 42.8% of the interviewed sample received the sticker as sign for their commitment to construct a latrine, and a further 12.7% received the flag as a sign of their completed latrine. These figures are rather poor compared to the participation rates in the CLTS triggering event (66.8% for CLTS only). The additional RANAS-based activities might therefore produce greater success if implemented more diligently. Another reason that we did not observe additional effects for the RANAS interventions may be a ceiling effect. As classic CLTS alone promoted latrine construction very powerfully, the RANAS interventions were not able to add to its effectiveness. It remains an open question whether the RANAS interventions would work well if implemented prior to CLTS, or as a stand-alone intervention.

This study is the first to show that changes in people's mindsets are responsible for the intervention effects of CLTS on latrine construction. Following our hypothesis, positive changes in psychosocial determinants caused by participation in CLTS led to higher latrine coverage in the communities.

CLTS made participants more aware of the latrine construction behaviour of their social environment and led to an increased perception that community leaders approve of latrine construction. Participants in CLTS arms compared to controls developed greater confidence that they would be able to construct, maintain, and repair their own household latrine. They were more committed and more likely to form action plans detailing how, when, and with whose help to construct latrines. These changes were all positively related to a higher probability of constructing latrines.

The relevance and effectiveness of changes in social norms for latrine construction, as in our case with others' behaviour and others' approval, have been reported by previous research (Bongartz et al., 2016; Dooley et al., 2016; Harter et al., 2018; Venkataramanan et al., 2018). CLTS was also able to make people feel more confident in constructing and maintaining their own household latrine, and this higher confidence helped them to actually complete this task. This finding is in line with previous research on WASH behaviours—for example, in the case of Burundi (Sonego and Mosler, 2014). In our study, CLTS also achieved its goal by strengthening peoples’ commitment to latrine construction. Commitment is the driver that transforms a plan into action and, within the RANAS model, is therefore located in the self-regulation factor block (Mosler, 2012). The role of commitment as an important predictor of hygienic behaviour has been shown by previous research in the WASH sector (e.g., for hand-washing in Ethiopia) (Contzen and Inauen, 2015).

Our results further imply that individuals within the same community react differently on the intervention. The changes in psychosocial determinants varied within communities, which implies that tailoring interventions to individual's needs might even increase its success. Of course, this idea stands in contrast to practical considerations of time and financial aspects.

### 4.1 Practical implications

The results of the mediation analyses imply that CLTS is already successful and further showed that the effects of CLTS were achieved through changes on several psychosocial determinants that were previously identified as key variables for latrine construction. Both might explain why additional RANAS-based intervention activities did not improve CLTS′ effectiveness significantly. Yet, despite high increases in latrine coverages, communities hardly reached 70%, which means that it can still be improved to reach full coverages and stop open defecation radically. The implementation of stand-alone theory-based interventions developed based on the RANAS approach might have achieved similar or even superior effects, but this assumption was not tested here. The combination of CLTS with theory-based public commitment, together with stickers and flags for commitment and completion of a latrine, was slightly more effective in increasing latrine coverage but was not statistically significant (higher increase of 8.9%). CLTS was successful because of changes on psychosocial determinants. In order to improve CLTS, practitioners might intensify those BCTs that are supposed to have led to the relevant changes on psychosocial determinants. Those BCTs were based on the RANAS catalogue of BCTs (Mosler and Contzen, 2016) and included those that strengthened the norm factors: The selection of natural leaders that serve as role models should be maintained in order to strengthen the commitment and personal importance of constructing latrines (BCT 14). Natural leaders further show the level of acceptance and approval of latrine construction and therefore strengthen the factor other's approval (BCT 11). The implementation of community action plans further helped to make the behaviour of others visible to the whole community (BCT 9). Besides norm factors, action plans improved participants abilities to define goals and develop steps towards latrine construction (BCT 26). During the triggering event and follow-up visits, latrine options were discussed, technical support was given, and questions concerning the construction process were answered (BCT 18).

### 4.2 Strengths, limitations, and conclusions

The study has several limitations. All data concerning psychosocial determinants and outcome variables were self-reported. An objective measure for latrine construction was gained through the observations of the data collectors. This method showed strong agreement between observed and self-reported latrine construction, so the self-reported data was used.

Another limitation concerns conclusions about the nature of the mediating effects. Due to the experimental design, causal conclusions on the changes in psychosocial determinants are likely. Yet, the causal nature of the relationship between changes in psychosocial factors and latrine constructions cannot be established with our data. In our study, the psychosocial determinants and latrine construction were both measured at the same time. Assessing psychosocial determinants before latrine construction may strengthen causal conclusions somewhat. But, more experimental research (e.g. manipulating the determinants others’ behaviour and vulnerability to test effects on latrine construction) is ultimately needed to provide conclusive tests of the causality of these relationships. Moreover, the outcome measure, latrine construction, included both completed latrines and those still being under construction. The reason for this decision was that the first follow-up visit was realized only some months after CLTS implementation, whereas the completion of latrines may take longer. The analysis of the final follow-up survey and the consideration of the percentage of CLTS participants having stopped open defecation will serve as a more robust outcome measure.

The success of CLTS in this study only accounts for the way CLTS was implemented by Global Communities for this project. The CLTS approach is meant to be adapted to local conditions and needs and therefore shows great variation between implementing organizations.

The study also has several strengths. This project is one of the first fully-powered cluster-randomized trials on CLTS. It therefore provides strong evidence for the success of CLTS. With 25 clusters and 625 individuals on average for each of the five intervention arms, the sample size of 3216 households allows unusually robust and reliable statistical analysis. Multilevel analysis was able to reveal and best account for the heterogeneity between and within communities. Cluster randomization is another strength that serves the external validity of our findings and prevents community differences interfering with intervention effects. In this research project, CLTS was implemented in a variety of community and physical contexts. The success of CLTS across all the conditions within this study offers strong encouragement to scale implementation up to other regions of Ghana and even to other countries in West Africa.

In addition, this study was the first to examine the psychosocial mechanisms of CLTS. The need to examine in greater detail the mechanisms of behaviour change interventions has recently been emphasized by the National Institutes of Health (Nielsen et al., 2017). As our study demonstrates, these results can provide important insights into how an intervention works and how it can be improved. Analyses of psychosocial determinants that lead to behaviour change therefore serves as a potential basis to improve the sanitation situation of rural communities.

## Data statement

The data are available on request with first author (data statement).

## Authorship contributions

Are needed for this article. Please request these from the authors, describing the role of each author in this article and the research it reports.
