# Supplementary material for: How does Community-Led Total Sanitation (CLTS) promote latrine construction, and can it be improved? A cluster-randomized controlled trial in Ghana
Source: Soc Sci Med. 2020 Jan;245:112705. doi: 10.1016/j.socscimed.2019.112705 (PMC6983942; doi:10.1016/j.socscimed.2019.112705)
Supplement: Multimedia component 1 [file mmc1.docx]

**Figure S1.** Visual scale for 5-point Likert-type answer options

### Table S1. Behaviour change techniques selection and intervention description

| **Intervention** | **RANAS determinants targeted ^a^** | **BCT description strategies** | **BCTs based on Michie et al. behaviour change taxonomy ^b^ or based on Abraham and Kools ^c^** |
| --- | --- | --- | --- |
| **CLTS** | Health knowledge | Facilitate the open defecation map with location of houses and places for open defecation. Discuss the fecal-oral transmission route and scenarios of the daily life of participants, e.g., food preparation, water collection and open defecation and the ways of possible transmission of faces to the household by e.g., chicken, dogs or pigs. | 5.1 Information about health consequences ^b^ |
|  | Vulnerability | Localization of the respondent’s household and open defecation areas on the community map to assess personal health risks. | 2.2 Feedback on behavior  5.2 Salience of consequences ^b^ |
|  | Severity | Calculate the medical costs of diarrheal illness and costs of losses in work power, comparison of costs of a toilet to costs for medical consultation. | 6 Emphasize severity of negative consequences to arouse fear ^c^ |
|  | Beliefs about costs and benefits | Discussion of different latrine types and construction possibilities and access to materials as well as encouraging the construction with local materials. | 2.2 Feedback on behavior  5.2 Salience of consequences ^b^ |
|  | Confidence in performance | Provision of answers to questions concerning latrine construction and technical support to help people gain confidence in their ability to construct latrines. | 4.1 Instruction on how to perform a behavior ^b^ |
|  | Personal importance/ Commitment | Identification of natural leaders who emerge during the triggering process. Strengthening their role as models for the community. | 13.1 Identification of self as role model ^b^ |
|  | Others’ (Dis) Approval | Natural leaders and the CLTS implementers clearly show that they approve of using a toilet and stopping open defecation. | 6.3 Information about others` approval ^b^ |
|  | Action Planning | Development of a **community** action plan for achieving the goal of an open defecation free community. Setting of an agreed date for assessment for verification. | 1.4 Action planning (including implementation intentions) ^b^ |
|  | Others’ behavior | By creating a community action plan, the whole community witnesses the number of others that are willing to start latrine construction. |  |
| **Action planning** | Beliefs about costs and benefits | Provide information about costs and benefits of different latrine options and discuss the individual (financial/ contextual) situation and possibilities. | 2 Describe likely material consequences of behavior  6 Describe emotional (or affective) consequences of behavior ^c^ |
|  | Confidence in recovery | In the case of setbacks during the latrine construction process, e.g., because pits collapse, assure community members that this is not their fault and that this can happen. Support them in finding a better site or latrine option. | 4.1 Instruction on how to perform a behavior ^b^ |
|  | Action Planning | Individual planning of when, how and with whose responsibility to construct an own household latrine. Definition of materials and latrine type for this specific household. Placing individual household action plan at the wall of the household. | 1.4 Action planning (including implementation intentions) ^b^ |
|  | Commitment | Signing the action plan for latrine construction by the facilitator and the responsible for latrine construction. | 1.8 Behavioral contract ^b^ |
| **Public commitment** | Feelings | Describe people owning latrines as well respected and advanced. | 5.6 Emotional consequences ^b^ |
|  | Others’ (dis) approval | Invite opinion leaders to stand in front of the community and pledge to be the first ones to construct a latrine. | 6.3 Information about others` approval ^b^ |
|  | Commitment | Let people publicly pledge to construct a latrine and note down the names. | 1.8 Behavioral contract ^b^ |
|  | Other’s behaviour | Let people show their commitment by placing a visible symbol at their house, when they started constructing a latrine. | 1.9 Commitment ^b^ |

*^a^ Mosler, H.-J. (2012). A systematic approach to behavior change interventions for the water and sanitation sector in developing countries: A conceptual model, a review, and a guideline. International Journal of Environmental Health Research, 22(5), 431–449.*

*^b^ Michie, S., Richardson, M., Johnston, M., Abraham, C., Francis, J., Hardeman, W., et al. (2013). The Behavior Change Technique Taxonomy (v1) of 93 Hierarchically Clustered Techniques: Building an International Consensus for the Reporting of Behavior Change Interventions. Annals of Behavioral Medicine, 46, 81-95.*

*^c^ Abraham, C., & Kools, M. (2011). Writing health communication: An evidence-based guide: Sage.*

**Table S2.** Items included for analysis

| Risk factor block | |  |  |  |
| --- | --- | --- | --- | --- |
|  | |  |  |  |
|  | Vulnerability ^a^ | Generally, how high do you think is the risk that you get diarrhoea? | 1=not at all high to 5=very high | score of 14 items relating to reasons, body effects and preventive measures for diarrhoea |
|  | Severity | Imagine that you have diarrhoea, how severe would be the impact on your life? | 1=not at all high to 5=very high |  |
|  | Health Knowledge | Can you tell me what causes diarrhoea? Could you please tell me for each following aspects whether it is a cause or not? |  |  |
|  |  | Food touched by an infected person | 1=Yes; 0=No; 99=I don't know |  |
|  |  | Contact with the saliva of an infected person | 1=Yes; 0=No; 99=I don't know |  |
|  |  | Shaking hands with an infected person | 1=Yes; 0=No; 99=I don't know |  |
|  |  | Water contaminated by bacteria | 1=Yes; 0=No; 99=I don't know |  |
|  |  | Defecate in the open | 1=Yes; 0=No; 99=I don't know |  |
|  |  | Flies touching the food | 1=Yes; 0=No; 99=I don't know |  |
|  |  | Could you please tell me for each whether it is a body effect of diarrhoea or not? |  |  |
|  |  | Cough | 1=Yes; 0=No; 99=I don't know |  |
|  |  | Loss of water and salt from the body | 1=Yes; 0=No; 99=I don't know |  |
|  |  | Fever | 1=Yes; 0=No; 99=I don't know |  |
|  |  | Three or more loose stools per day |  |  |
|  |  | Could you please tell me for each whether it is a preventive measure for diarrhoea or not? |  |  |
|  |  | Drinking treated water (e.g. chlorinated, filtered, boiled) | 1=Yes; 0=No; 99=I don't know |  |
|  |  | Using safe latrines for defecation | 1=Yes; 0=No; 99=I don't know |  |
|  |  | Drinking oral rehydration salt | 1=Yes; 0=No; 99=I don't know |  |
|  |  | Washing hands with soap after eating | 1=Yes; 0=No; 99=I don't know |  |
|  |  |  |  |  |

| Attitudes factor block | | |  |  |
| --- | --- | --- | --- | --- |
|  | Feelings | How proud are you of your own latrine? | 1=not at all proud to 5= very proud | α (T0) = 0.29  α (T1) = 0.28 |
|  |  | Do you think you are more respected by your community because you have your own latrine? | 1= not at all more respected to 5= very much more respected |  |
|  |  | If you construct a latrine, how vulnerable are you for envy? | 1=not at all to 5=very much |  |
|  | Beliefs about costs and benefits | How expensive do you think is it to construct your own latrine? | 1=not at all expensive to 5= very expensive | α (T0) = 0.39  α (T1) = 0.61 |
|  |  | How difficult is it to find the money to construct your own latrine? | 1=not at all difficult to 5= very difficult |  |
|  |  | How difficult is it to find the time and effort to construct your own latrine? | 1=not at all difficult to 5= very difficult |  |
|  |  |  |  |  |

| Norm factor block | |  |  |  |
| --- | --- | --- | --- | --- |
|  | Other's behavior | How many of your relatives within your community constructed their own latrines? | 1=(Almost) nobody (0%) to 5=(Almost) all of them (100%) | α (T0) = .72  α (T1) = 0.94 |
|  |  | How many members of your community constructed their own latrines? | 1=(Almost) nobody (0%) to 5=(Almost) all of them (100%) |  |
|  | Other's approval | How much do people who are important to you (e.g. family, parents, friends) approve that you construct a latrine? | 1=approve not at all to 5=approve very much | α (T0) = 0.75  α (T1) = 0.75 |
|  |  | People who are leaders in the community (e.g. opinion leader, Chief of village, etc.) how much do they encourage you to construct your own latrine? | 1=not at all to 5=very much |  |

| Abilities factor block | |  |  |  |
| --- | --- | --- | --- | --- |
|  | How-to-do-knowledge | Can you tell us for each of the following features if this is neccessary for a hygienically safe latrine? |  |  |
|  |  | paint the walls | 1=Yes; 0=No; 99=I don't know | score of 6 items |
|  |  | vent pipe | 1=Yes; 0=No; 99=I don't know |  |
|  |  | have seperate latrines for men and women | 1=Yes; 0=No; 99=I don't know |  |
|  |  | decking without holes (other than the drop hole) | 1=Yes; 0=No; 99=I don't know |  |
|  |  | weeding around the latrine | 1=Yes; 0=No; 99=I don't know |  |
|  |  | dark inside the latrine | 1=Yes; 0=No; 99=I don't know |  |
|  | Confidence in performance | How confident are you that you can construct a latrine even if this is difficult (e.g. gathering the materials)? | 1=not at all confident to 5=very confident | α (T0) = 0.68  α (T1) = 0.78 |
|  | Confidence in maintenance | How confident are you that you could finish the construction of a latrine even if problems arise (e.g. you run out of money)? | 1=not at all confident to 5=very confident |  |
|  | Confidence in recovery | Imagine that the latrine got damaged. How confident are you that you will be able to repair the latrine again? | 1=not at all confident to 5=very confident |  |

| Self-regulation factor block | |  |  |  |
| --- | --- | --- | --- | --- |
|  | Action planning | Do you have a plan when you will construct a latrine? | 1=Yes; 0=No | score of 5 items |
|  |  | Do you have a plan how you will gather the materials for the latrine construction? | 1=Yes; 0=No |  |
|  |  | Do you have a plan how you will get the money to buy the materials for the latrine construction? | 1=Yes; 0=No |  |
|  |  | Do you have a plan who will help you to construct the latrine? | 1=Yes; 0=No |  |
|  | Barrier planning | Do you have a plan how you can construct a latrine if you are running out of materials? | 1=Yes; 0=No |  |
|  | Commitment | How committed are you to construct a latrine? | 1=not at all committed to 5=very committed | α (T0) = 0.61  α (T1) = 0.66 |
|  | Personal obligation | How strongly do you feel a personal obligation to construct your own latrine? | 1=not at all to 5=very much |  |
|  | | | |  |

*Note:* Cronbach’s alpha reported for baseline and follow-up respectively. All values were later on transformed to range between 0 and 1
^a^ For single item measurement we refer to the C-OAR-SE Framework presented by Rossiter (2011)

**Table S3.** Descriptive measures, Intra-class correlation and correlation matrix for psychosocial determinants

|  | Mean | SD^a^ | Ρ (ICC)^b^ | 1 | 2 | 3 | 4 | 5 | 6 | 7 | 8 | 9 | 10 | 11 | 12 | | VIF |
| --- | --- | --- | --- | --- | --- | --- | --- | --- | --- | --- | --- | --- | --- | --- | --- | --- | --- |
| Latrine Ownership |  |  |  |  |  |  |  |  |  |  |  |  |  |  |  | |  |
| Vulnerability | -0.06 | 0.46 | 0.04 | -0.06** |  |  |  |  |  |  |  |  |  |  |  | | 1.12 |
| Severity | 0.06 | 0.32 | 0.07 | 0.04 | .24** |  |  |  |  |  |  |  |  |  |  | | 1.10 |
| Health knowledge | -0.02 | 0.16 | 0.04 | -0.08** | .05** | -.02 |  |  |  |  |  |  |  |  |  | | 1.03 |
| Feelings | 0.06 | 0.23 | 0.06 | -0.10** | .10** | .09** | .00 |  |  |  |  |  |  |  |  | | 1.08 |
| Beliefs about costs/benefits | 0.01 | 0.29 | 0.05 | 0.09** | .02 | .03 | -.04 | .15** |  |  |  |  |  |  |  | | 1.05 |
| Other’s behaviour | 0.42 | 0.44 | 0.65 | 0.67** | -.09** | .03 | -.09** | -.11** | -.09** |  |  |  |  |  |  | | 1.42 |
| Other’s (dis)approval | 0.28 | 0.42 | 0.14 | 0.21** | .03 | .10** | -.09** | .07** | .06** | .27** |  |  |  |  |  | | 1.17 |
| How-to-do-knowledge | 0.12 | 0.25 | 0.19 | 0.08** | .08** | .11** | -.03 | .01 | -.02 | .10** | .02 |  |  |  |  | | 1.04 |
| Self-efficacy | 0.18 | 0.35 | 0.06 | 0.18** | .15** | .03 | -.07** | .13** | .01 | .21** | .21** | .08** |  |  |  | | 1.27 |
| Commitment/pers. norm | 0.08 | 0.28 | 0.10 | 0.15** | .10** | .15** | -.06** | .11** | .07** | .17** | .23** | .09** | .35** |  |  | | 1.23 |
| Action Planning | 0.33 | 0.44 | 0.34 | 0.65** | .00 | .03 | -.05** | -.02 | -.09** | .47** | .21** | .14** | .25** | .19** |  | | 1.39 |
| Barrier Planning | 0.13 | 0.48 | 0.02 | 0.05 | .06** | .06** | .05** | .08** | -.02 | .04 | .02 | .08** | .18** | .12** | .18** | | 1.08 |
| Significance levels: **p < 0.01. Determinants are differences baseline to follow-up (grand-mean cantered).  ^a^ *SD*= standard deviation, ^b^  *Ρ* *(ICC)*=Intra-class correlation | | | | | | | | | | | | | | | |  |  |

**Table S4.** Coding for main effect analysis of CLTS compared to other intervention arms and control arm.

|  | Any CLTS | CLTS+RANAS_Com | CLTS+RANAS_Plan | CLTS+RANAS_ComPlan |
| --- | --- | --- | --- | --- |
| Coding  Model effects | 0=yes; 1=no | 0=no; 1=yes | 0=no; 1=yes | 0=no; 1=yes |
| Intercept (CLTS effect only) | 0 | 0 | 0 | 0 |
| CLTS-only: Control arm vs. CLTS-only | 1 | 0 | 0 | 0 |
| CLTS+RANAS_Com: RANAS with Public Commitment vs. CLTS-only | 0 | 1 | 0 | 0 |
| CLTS+RANAS_Plan: RANAS with Action Planning vs. CLTS-only | 0 | 0 | 1 | 0 |
| CLTS+RANAS_ComPlan: RANAS with Public Commitment and Action Planning vs. CLTS-only | 0 | 0 | 0 | 1 |

**Table S5.** Descriptive baseline measures for dropouts and respondents remaining in the sample at follow-up

|  | Dropouts | Respondents | *Cramer's V* | *p* |  |
| --- | --- | --- | --- | --- | --- |
| *n* | 512 | 2704 |  |  |  |
| Occupation |  |  | 0.02 | 0.202 |  |
| farming | 78.3% | 80.8% |  |  |  |
| other (trading, mining, fishing) | 21.7% | 19.2% |  |  |  |
| Religion |  |  | 0.09 | 0.108 |  |
| Islam | 23.0% | 25.1% |  |  |  |
| Christian | 35.8% | 47.1% |  |  |  |
| Traditional religion | 11.3% | 17.9% |  |  |  |
| Atheists | 11.3% | 5.2% |  |  |  |
| Female respondents | 41.0% | 42.9% | 0.01 | 0.438 |  |
| Latrine construction | 3.9% | 3.0% | 0.02 | 0.301 |  |
|  |  |  |  |  |  |
|  | Mean *(SD)* | Mean *(SD)* | F | *p* | *d* |
| Age | 36.84 *(15.82)* | 43.51 *(16.12)* | 37.22 | <.001 | 0.22 |
| Income | 252.76 *(473.30)* | 192.73 *(359.40)* | 10.71 | .001 | 0.12 |
| Household size | 8.53 *(4.91)* | 8.73 *(4.84)* | 0.74 | 0.389 | 0.03 |
| Education | 6.49 *(7.63)* | 2.69 *(4.69)* | 2.23 | .135 | 0.05 |

*Note:* Effect sizes for independent means according to Cohen (1992): *d*=.2 (small), *d*=.5 (medium), *d*=.8 (large) and for Cramer’s *V*: *V*=.1 (small), *V*=.3 (medium), *V*=.5 (large) (Ferguson, 2009).

**Table S6.** Results of multiple imputation

|  | **Drop-Outs** | | | | | | | | | | **Missing values** |  |  | |
| --- | --- | --- | --- | --- | --- | --- | --- | --- | --- | --- | --- | --- | --- | --- |
|  |  | | | | | Number of missing values | | Percent of missingness | | |  | Number of missing values | Percent of missingness | |
| Missingness | **Number of complete drop-outs**  **Reasons for drop-outs:**  Passed away  Permanently left community (e.g., married outside, left for work)  Currently travelled (e.g., visiting relatives, business)  other reasons (e.g., could not be identified, currently at the market) | | | | 512  66  145  251  50 | | | 100%  12.9%  28.3%  49.0%  9.8% | | | Latrine construction (outcome)  M1: Vulnerability  M2: Severity  M3: Factual Knowledge  M4: Affective Beliefs  M5: Instrumental Beliefs  M6: Descriptive Norms  M7: Injunctive Norms  M8: Action Knowledge  M9: Self-Efficacy  M10: Commitment /Personal Norm  M11: Action Planning  M12: Coping Planning | 1  1  0  0  3  0  0  3  0  1  0  0  2 | | 0.0%  0.0%  0.1%  0.0%  0.0%  0.1%  0.0%  0.0%  0.1% |
| Pattern of missingness | **MNAR:** drop-out depending on age and income (see Table S4) | | | | | | | | | | **MCAR:** Little’s MCAR Test: *X^2^* (55)= 70.28, *p=*0.08 | | | |
| Used software | Multiple imputation (IBM SPSS Statistics 24) | | | | | | | | | | | | | |
| Imputation model | Imputation method  Iterations  Model for scale variables  Number of imputations | | Fully Conditional Specification  10  Linear Regression  5 | | | | | | | | | | | |
|  | | | | | | | | | | | | | | |
|  | | Imputation model  M3: Factual Knowledge  M8: Action Knowledge  M11: Action Planning  Latrine Construction  M1: Vulnerability  M2: Severity  M5: Instrumental Beliefs  M6: Descriptive Norms  M10: Commitment /Personal Norm  M9: Self-Efficacy  M12: Coping Planning  M4: Affective Beliefs  M7: Injunctive Norms | | Type  Lin.Reg.  Lin.Reg.  Lin.Reg.  Log.Reg.  Lin.Reg.  Lin.Reg.  Lin.Reg.  Lin.Reg.  Lin.Reg.  Lin.Reg.  Lin.Reg.  Lin.Reg.  Lin.Reg. | | | Effects  Intervention conditions and mediators | | Missing values/ Imputed values  512/2560  512/2560  512/2560  513/2565  513/2565  513/2565  513/2565  513/2565  513/2565  514/2570  514/2570  516/2580  516/2580 |  | | | | |
| Interpretation | | Imputed datasets were used for analysis of main effects. No changes in main effects were observed. | | | | | | | | | | | | |
| Sensitivity analysis | | Main effects were calculated with additional consideration of baseline covariates (see main text, Table 2). No differences in main effects were observed. | | | | | | | | | | | | |

###

### Table S7. Results of single multilevel mediations of intervention effect on latrine construction mediated by changes in RANAS-based psychosocial determinants

|  | |  | | CLTS intervention (a-path) | | | | | Latrine construction (b-path) | | | | | Indirect effects (a*b-path) | | |
| --- | --- | --- | --- | --- | --- | --- | --- | --- | --- | --- | --- | --- | --- | --- | --- | --- |
|  | |  | |  |  |  | CI95 |  |  |  |  | CI95 |  |  | CI95 |  |
|  | |  | | Estimate *(SE)* | p | OR | LL | UL | Estimate *(SE)* | p | OR | LL | UL | Estimate *(SE)* | LL | UL |
|  | |  | |  |  |  |  |  |  |  |  |  |  |  |  |  |
| **Vulnerability** | | | |  |  |  |  |  |  |  |  |  |  | 0.12 (0.20) | -0.27 | 0.51 |
|  | fixed intercept | | | 0.01 (0.03) | 0.643 | 1.01 | 0.96 | 1.07 | 4.09 *(0.25)* | 0.000 | 59.80 | 36.20 | 98.79 |  |  |  |
|  | fixed effect | | | -0.02 *(0.03)* | 0.546 | 0.98 | 0.92 | 1.05 | -6.43 *(1.73)* | 0.000 | 0.00 | 0.00 | 0.05 |  |  |  |
|  | random intercept | | | <0.01 (<0.01) | 0.000 |  |  |  | 3.55 (0.69) | 0.000 |  |  |  |  |  |  |
|  | random effect (level 2) | | |  |  |  |  |  | 0.98 (0.56) | 0.078 |  |  |  |  |  |  |
|  | residual variance (level 1) | | |  |  |  |  |  | 0.20 (<0.01) | 0.000 |  |  |  |  |  |  |
| **Severity** | | | |  |  |  |  |  |  |  |  |  |  | <-0.01 *(0.21)* | -0.42 | 0.42 |
|  | | fixed intercept | | <0.01 *(0.03)* | 0.921 | 1.00 | 0.94 | 1.07 | 4.16 (0.46) | 0.000 | 64.20 | 25.38 | 162.39 |  |  |  |
|  | | fixed effect | | <0.01 *(0.03)* | 0.992 | 1.00 | 0.93 | 1.07 | 6.36 (2.79) | 0.023 | 576.51 | 2.17 | 152817.55 |  |  |  |
|  | | random intercept | | <0.01 (<0.01) | 0.002 |  |  |  | 3.46 (0.68) | 0.000 |  |  |  |  |  |  |
|  | | random effect (level 2) | |  |  |  |  |  | 1.12 (0.47) | 0.000 |  |  |  |  |  |  |
|  | | residual variance (level 1) | | |  |  |  |  | 0.09 (<0.01) | 0.000 |  |  |  |  |  |  |
| **Health Knowledge** | | | |  |  |  |  |  |  |  |  |  |  | 0.91 *(1.6)* | -2.23 | 4.05 |
|  | | fixed intercept | | 0.02 (0.04) | 0.596 | 1.02 | 0.95 | 1.10 | 3.37 (4.24) | 0.000 | 28.93 | 0.01 | 139385.67 |  |  |  |
|  | | fixed effect | | -0.03 (0.04) | 0.487 | 0.97 | 0.90 | 1.05 | -34.09 *(11.03)* | 0.427 | 0.00 | 0.00 | 0.00 |  |  |  |
|  | | random intercept | | <0.01 (<0.01) | 0.499 |  |  |  | 2.94 (1.42) | 0.000 |  |  |  |  |  |  |
|  | | random effect (level 2) | |  |  |  |  |  | 2.33 (8.37) | 0.781 |  |  |  |  |  |  |
|  | | residual variance (level 1) | |  |  |  |  |  | 0.02 (<0.01) | 0.000 |  |  |  |  |  |  |
| **Feelings** | | | |  |  |  |  |  |  |  |  |  |  | 1.07 (0.58) | -0.07 | 2.22 |
|  | | fixed intercept | | 0.04 (0.01) | 0.01 | 1.04 | 1.01 | 1.07 | 4.03 (0.30) | 0.000 | 56.26 | 30.88 | 102.51 |  |  |  |
|  | | fixed effect | | -0.05 *(0.01)* | 0.000 | 0.95 | 0.92 | 0.98 | -21.20 *(11.91)* | 0.075 | 0.00 | 0.00 | 13.82 |  |  |  |
|  | | random intercept | | <0.01 (<0.01) | 0.000 |  |  |  | 2.95 (0.90) | 0.000 |  |  |  |  |  |  |
|  | | random effect (level 2) | |  |  |  |  |  | 7.46 (2.26) | 0.000 |  |  |  |  |  |  |
|  | | residual variance (level 1) | |  |  |  |  |  | 0.05 (<0.01) | 0.000 |  |  |  |  |  |  |
| **Beliefs about costs and benefits** | | | | |  |  |  |  |  |  |  |  |  | 0.25 (1.06) | -1.83 | 2.33 |
|  | | fixed intercept | 0.03 (0.02) | | 0.142 | 1.03 | 0.99 | 1.07 | 4.22 (2.32) | 0.069 | 68.24 | 0.66 | 7079.79 |  |  |  |
|  | | fixed effect | -0.04 *(0.02)* | | 0.087 | 0.96 | 0.92 | 1.01 | -6.25 (27.44) | 0.820 | <0.01 | <0.01 | 18.76 |  |  |  |
|  | | random intercept | <0.01 (<0.01) | | 0.000 |  |  |  | 3.41 (1.08*)* | 0.000 |  |  |  |  |  |  |
|  | | random effect (level 2) |  | |  |  |  |  | 4.36 (9.76) | 0.655 |  |  |  |  |  |  |
|  | | residual variance (level 1) |  | |  |  |  |  | 0.08 (<0.01) | 0.000 |  |  |  |  |  |  |
| **Others’ behaviour** | | |  | |  |  |  |  |  |  |  |  |  | 1.75 (0.34) | 1.08 | 2.42 |
|  | | fixed intercept | -0.15 *(0.04)* | | 0.000 | 0.86 | 0.80 | 0.93 | 1.38 (0.91) | 0.130 | 0.39 | 0.29 | 0.53 |  |  |  |
|  | | fixed effect | 0.28 (0.05) | | 0.000 | 1.33 | 1.21 | 1.45 | 6.18 (0.45) | 0.000 | 300.07 | 114.66 | 785.25 |  |  |  |
|  | | random intercept | 0.09 (<0.01) | | 0.000 |  |  |  | 0.52 (0.35) | 0.145 |  |  |  |  |  |  |
|  | | random effect (level 2) |  | |  |  |  |  | 0.30 (0.16) | 0.051 |  |  |  |  |  |  |
|  | | residual variance (level 1) |  | |  |  |  |  | 0.07 (<0.01) | 0.000 |  |  |  |  |  |  |
| **Others’ approval** | | |  | |  |  |  |  |  |  |  |  |  | 1.72 (1.02) | 0.27 | 3.72 |
|  | | fixed intercept | -0.10 *(0.03)* | | 0.001 | 0.91 | 0.85 | 0.96 | 2.42 (0.95) | 0.000 | 11.25 | 1.69 | 74.74 |  |  |  |
|  | | fixed effect | 0.15 (0.04) | | 0.000 | 1.16 | 1.08 | 1.24 | 11.84 *(4.76)* | 0.013 | 138136.83 | 10.24 | 1864017608.90 |  |  |  |
|  | | random intercept | 0.02 (<0.01) | | 0.001 |  |  |  | 2.57 (0.86) | 0.000 |  |  |  |  |  |  |
|  | | random effect (level 2) |  | |  |  |  |  | 0.73 (1.63) | 0.655 |  |  |  |  |  |  |
|  | | residual variance (level 1) |  | |  |  |  |  | 0.16 (<0.01) | 0.000 |  |  |  |  |  |  |
| **How-to-do-knowledge** | | | | |  |  |  |  |  |  |  |  |  | 0.13 (0.11) | -0.10 | 0.35 |
|  | | fixed intercept | -0.03 *(0.03)* | | 0.282 | 0.97 | 0.91 | 1.03 | 3.63 (0.25) | 0.000 | 37.64 | 22.65 | 62.55 |  |  |  |
|  | | fixed effect | 0.04 (0.03) | | 0.243 | 1.04 | 0.97 | 1.11 | 3.25 (1.73) | 0.061 | 25.82 | 0.81 | 824.68 |  |  |  |
|  | | random intercept | 0.01 (<0.01) | | 0.000 |  |  |  | 3.54 (0.63) | 0.000 |  |  |  |  |  |  |
|  | | random effect (level 2) |  | |  |  |  |  | 0.41 (0.27) | 0.127 |  |  |  |  |  |  |
|  | | residual variance (level 1) |  | |  |  |  |  | 0.05 (<0.01) | 0.000 |  |  |  |  |  |  |
| **Confidence in performance/ maintenance/ recovery** | | | | | | |  |  |  |  |  |  |  | 1.75 (0.69) | 0.39 | 3.10 |
|  | | fixed intercept | -0.09 *(0.02)* | | 0.000 | 0.92 | 0.89 | 0.95 | 3.95 (1.41) | 0.000 | 51.99 | 3.08 | 877.43 |  |  |  |
|  | | fixed effect | 0.12 (0.02) | | 0.000 | 1.12 | 1.08 | 1.17 | 15.22 (4.45) | 0.001 | 4057185.24 | 550.04 | 29926194378.75 |  |  |  |
|  | | random intercept | 0.01 (<0.01) | | 0.001 |  |  |  | 2.72 (0.70) | 0.000 |  |  |  |  |  |  |
|  | | random effect (level 2) |  | |  |  |  |  | 0.96 (6.39) | 0.881 |  |  |  |  |  |  |
|  | | residual variance (level 1) |  | |  |  |  |  | 0.11 (<0.01) | 0.000 |  |  |  |  |  |  |
| **Commitment** | | |  | |  |  |  |  |  |  |  |  |  | 0.67 (0.43) | -0.18 | 1.52 |
|  | | fixed intercept | -0.06 *(0.03)* | | 0.05 | 0.94 | 0.89 | 1.00 | 4.00 (0.52) | 0.000 | 54.38 | 19.30 | 153.24 |  |  |  |
|  | | fixed effect | 0.08 (0.04) | | 0.041 | 1.08 | 1.00 | 1.17 | 8.58 (3.12) | 0.006 | 5340.10 | 10.52 | 2711465.98 |  |  |  |
|  | | random intercept | 0.01 (<0.01) | | 0.006 |  |  |  | 3.16 (0.67) | 0.000 |  |  |  |  |  |  |
|  | | random effect (level 2) |  | |  |  |  |  | 2.15 (3.27) | 0.511 |  |  |  |  |  |  |
|  | | residual variance (level 1) |  | |  |  |  |  | 0.07 (<0.01) | 0.000 |  |  |  |  |  |  |
| **Action Planning** | | |  | |  |  |  |  |  |  |  |  |  | 6.03 (1.31) | 3.47 | 8.59 |
|  | | fixed intercept | -0.32 *(0.09)* | | 0.000 | 0.73 | 0.61 | 0.87 | 3.93 (0.55) | 0.000 | 50.86 | 16.83 | 153.70 |  |  |  |
|  | | fixed effect | 0.43 (0.09) | | 0.000 | 1.53 | 1.27 | 1.85 | 14.20 (0.34) | 0.000 | 1468864.19 | 738222.09 | 2922646.21 |  |  |  |
|  | | random intercept | 0.03 (<0.01) | | 0.000 |  |  |  | 3.11 (0.77) | 0.000 |  |  |  |  |  |  |
|  | | random effect (level 2) |  | |  |  |  |  | 1.77 (1.33) | 0.175 |  |  |  |  |  |  |
|  | | residual variance (level 1) |  | |  |  |  |  | 0.13 (<0.01) | 0.000 |  |  |  |  |  |  |
| **Barrier Planning** | | |  | |  |  |  |  |  |  |  |  |  | 0.10 (0.29) | -0.47 | 0.68 |
|  | | fixed intercept | -0.01 *(0.04)* | | 0.769 | 0.99 | 0.92 | 1.06 | 4.06 (1.33) | 0.000 | 0.31 | 0.04 | 2.19 |  |  |  |
|  | | fixed effect | 0.01 (0.04) | | 0.722 | 1.01 | 0.94 | 1.09 | 7.64 (3.34) | 0.022 | 788.40 | 0.00 | 150616092121.47 |  |  |  |
|  | | random intercept | <0.01 (<0.01) | | 0.189 |  |  |  | 3.35 (0.76) | 0.000 |  |  |  |  |  |  |
|  | | random effect (level 2) |  | |  |  |  |  | 0.47 (0.23) | 0.036 |  |  |  |  |  |  |
|  | | residual variance (level 1) |  | |  |  |  |  | 0.22 (<0.01) | 0.000 |  |  |  |  |  |  |

*Note:* Level 1: individuals, level 2: communities. *SE=* standard error. *OR=* Odds Ratio. *CI*=confidence interval.

**Table S8.** Results of multiple mediations of intervention effect on latrine construction mediated by changes in RANAS-based psychosocial determinants for each intervention group compared to control group

| Control vs. CLTS_only | Intervention (a-path) | |  |  |  | Latrine construction (b-path) | | | | | Indirect effects (a*b-path) | |  |
| --- | --- | --- | --- | --- | --- | --- | --- | --- | --- | --- | --- | --- | --- |
|  |  |  |  | CI_95_ |  |  |  |  | CI_95_ |  |  | CI_95_ |  |
|  | Estimate *(SE)* | *p* | OR | LL | UL | Estimate *(SE)* | *p* | OR | LL | UL | Estimate *(SE)* | LL | UL |
| M1: Vulnerability | -0.01 *(0.03)* | 0.668 | -0.43 | -0.06 | 0.04 | -0.47 *(0.16)* | 0.004 | -2.92 | -0.79 | -0.16 | 0.01 *(0.01)* | -0.02 | 0.04 |
| M2: Severity | 0.01 *(0.02)* | 0.472 | 0.72 | -0.02 | 0.05 | 0.36 *(0.24)* | 0.132 | 1.51 | -0.11 | 0.82 | 0.00 *(0.01)* | -0.01 | 0.03 |
| M3: Factual Knowledge | -0.01 *(0.01)* | 0.097 | -1.66 | -0.03 | 0.00 | -0.9 *(0.61)* | 0.138 | -1.48 | -2.10 | 0.29 | 0.01 *(0.01)* | 0.00 | 0.05 |
| M4: Affective Beliefs | -0.06 *(0.01)* | <0.01 | -4.88 | -0.09 | -0.04 | -0.85 *(0.45)* | 0.057 | -1.90 | -1.73 | 0.03 | 0.05 *(0.03)* | 0.00 | 0.12 |
| M5: Instrumental Beliefs | -0.05 *(0.02)* | 0.002 | -3.13 | -0.08 | -0.02 | 0.17 *(0.35)* | 0.636 | 0.47 | -0.52 | 0.86 | -0.01 *(0.02)* | -0.05 | 0.03 |
| M6: Descriptive Norms | 0.53 *(0.02)* | <0.01 | 29.91 | 0.49 | 0.56 | 3.09 *(0.30)* | <0.01 | 10.21 | 2.50 | 3.68 | 1.63 *(0.18)* | 1.29 | 1.98 |
| M7: Injunctive Norms | 0.21 *(0.02)* | <0.01 | 8.52 | 0.16 | 0.26 | -0.19 *(0.24)* | 0.426 | -0.80 | -0.65 | 0.28 | -0.04 *(0.05)* | -0.13 | 0.05 |
| M8: Action Knowledge | 0.04 *(0.01)* | 0.003 | 2.98 | 0.01 | 0.07 | -0.66 *(0.41)* | 0.106 | -1.61 | -1.46 | 0.14 | -0.03 *(0.02)* | -0.08 | 0.00 |
| M9: Self-Efficacy | 0.10 *(0.02)* | <0.01 | 4.99 | 0.06 | 0.14 | -0.26 *(0.30)* | 0.384 | -0.87 | -0.85 | 0.33 | -0.03 *(0.03)* | -0.09 | 0.03 |
| M10: Commitment/ Pers. Norm | 0.08 *(0.02)* | <0.01 | 5.11 | 0.05 | 0.11 | 0.02 *(0.40)* | 0.951 | 0.06 | -0.77 | 0.82 | 0.00 *(0.03)* | -0.06 | 0.06 |
| M11: Action Planning | 0.41 *(0.02)* | <0.01 | 18.99 | 0.37 | 0.45 | 3.81 *(0.32)* | <0.01 | 12.05 | 3.19 | 4.43 | 1.56 *(0.15)* | 1.27 | 1.86 |
| M12: Coping Planning | 0.02 *(0.03)* | 0.446 | 0.76 | -0.03 | 0.08 | -0.69 *(0.22)* | 0.002 | -3.10 | -1.12 | -0.25 | -0.01 *(0.02)* | -0.06 | 0.02 |
|  |  |  |  |  |  |  |  |  |  |  |  |  |  |
| Control vs. CLTS+RANAS_Com | Intervention (a-path) | |  |  |  | Latrine construction (b-path) | | | | | Indirect effects (a*b-path) | | |
|  |  |  |  | CI_95_ |  |  |  |  | CI_95_ |  |  | CI_95_ |  |
|  | Estimate *(SE)* | *p* | OR | LL | UL | Estimate *(SE)* | *p* | OR | LL | UL | Estimate *(SE)* | LL | UL |
| M1: Vulnerability | -0.01 *(0.03)* | 0.664 | -0.43 | -0.07 | 0.04 | -0.17 *(0.19)* | 0.352 | -0.93 | -0.54 | 0.19 | 0.00 *(0.01)* | -0.01 | 0.03 |
| M2: Severity | 0.03 *(0.02)* | 0.111 | 1.59 | -0.01 | 0.07 | 0.46 *(0.28)* | 0.102 | 1.63 | -0.09 | 1.01 | 0.01 *(0.01)* | 0.00 | 0.06 |
| M3: Factual Knowledge | -0.04 *(0.01)* | <0.01 | -3.95 | -0.05 | -0.02 | -0.12 *(0.67)* | 0.856 | -0.18 | -1.44 | 1.20 | 0.00 *(0.02)* | -0.04 | 0.06 |
| M4: Affective Beliefs | -0.06 *(0.01)* | <0.01 | -4.39 | -0.09 | -0.03 | -0.09 *(0.45)* | 0.835 | -0.21 | -0.98 | 0.79 | 0.01 *(0.03)* | -0.05 | 0.06 |
| M5: Instrumental Beliefs | -0.03 *(0.02)* | 0.067 | -1.84 | -0.07 | 0.00 | 0.22 *(0.36)* | 0.554 | 0.59 | -0.50 | 0.93 | -0.01 *(0.01)* | -0.05 | 0.01 |
| M6: Descriptive Norms | 0.58 *(0.02)* | <0.01 | 30.40 | 0.54 | 0.61 | 2.96 *(0.31)* | <0.01 | 9.39 | 2.34 | 3.57 | 0.17 *(0.20)* | 1.33 | 2.08 |
| M7: Injunctive Norms | 0.18 *(0.03)* | <0.01 | 7.23 | 0.13 | 0.23 | -0.28 *(0.26)* | 0.280 | -1.08 | -0.78 | 0.23 | -0.05 *(0.05)* | -0.15 | 0.04 |
| M8: Action Knowledge | 0.06 *(0.02)* | <0.01 | 3.93 | 0.03 | 0.09 | -0.09 *(0.42)* | 0.826 | -0.22 | -0.92 | 0.74 | -0.01 *(0.03)* | -0.06 | 0.05 |
| M9: Self-Efficacy | 0.09 *(0.02)* | <0.01 | 4.57 | 0.05 | 0.13 | -0.36 *(0.35)* | 0.299 | -1.04 | -1.05 | 0.32 | -0.03 *(0.03)* | -0.10 | 0.03 |
| M10: Commitment/ Pers. Norm | 0.08 *(0.02)* | <0.01 | 4.39 | 0.04 | 0.11 | 0.57 *(0.40)* | 0.160 | 1.40 | -0.22 | 1.36 | 0.04 *(0.03)* | -0.01 | 0.12 |
| M11: Action Planning | 0.44 *(0.02)* | <0.01 | 19.57 | 0.39 | 0.48 | 3.46 *(0.33)* | <0.01 | 10.33 | 2.80 | 4.11 | 1.51 *(0.16)* | 1.21 | 1.82 |
| M12: Coping Planning | 0.00 *(0.03)* | 0.906 | -0.12 | -0.06 | 0.05 | -0.27 *(0.22)* | 0.221 | -1.22 | -0.71 | 0.16 | 0.00 *(0.01)* | -0.02 | 0.03 |
|  |  |  |  |  |  |  |  |  |  |  |  |  |  |
| Control vs. CLTS+RANAS_Plan | Intervention (a-path) | |  |  |  | Latrine construction (b-path) | | | |  | Indirect effects (a*b-path) | | |
|  |  |  |  | CI_95_ |  |  |  |  | CI_95_ |  |  | CI_95_ |  |
|  | Estimate *(SE)* | *p* | OR | LL | UL | Estimate *(SE)* | *p* | OR | LL | UL | Estimate *(SE)* | LL | UL |
| M1: Vulnerability | -0.06 *(0.03)* | 0.034 | -2.12 | -0.11 | 0.00 | -0.45 *(0.02)* | 0.021 | -2.30 | -0.83 | -0.07 | 0.03 *(0.02)* | 0.00 | 0.07 |
| M2: Severity | -0.04 *(0.02)* | 0.049 | -1.97 | -0.08 | 0.00 | -0.15 *(0.27)* | 0.573 | -0.56 | -0.68 | 0.38 | 0.01 *(0.01)* | -0.01 | 0.04 |
| M3: Factual Knowledge | -0.01 *(0.01)* | 0.476 | -0.71 | -0.02 | 0.01 | -0.05 *(0.69)* | 0.942 | -0.07 | -1.40 | 1.30 | 0.00 *(0.01)* | -0.01 | 0.02 |
| M4: Affective Beliefs | -0.05 *(0.01)* | <0.01 | -3.67 | -0.08 | -0.02 | -0.44 *(0.48)* | 0.365 | -0.91 | -1.38 | 0.51 | 0.02 *(0.03)* | -0.02 | 0.08 |
| M5: Instrumental Beliefs | -0.05 *(0.02)* | 0.007 | -2.70 | -0.08 | -0.01 | 0.50 *(0.38)* | 0.179 | 1.34 | -0.23 | 1.24 | -0.02 *(0.02)* | -0.08 | 0.01 |
| M6: Descriptive Norms | 0.48 *(0.02)* | <0.01 | 26.83 | 0.44 | 0.51 | 2.98 *(0.36)* | <0.01 | 8.16 | 2.26 | 3.69 | 1.42 *(0.19)* | 1.05 | 1.78 |
| M7: Injunctive Norms | 0.16 *(0.03)* | <0.01 | 6.40 | 0.11 | 0.21 | -0.05 *(0.29)* | 0.876 | -0.16 | -0.61 | 0.52 | -0.01 *(0.05)* | -0.09 | 0.08 |
| M8: Action Knowledge | 0.03 *(0.02)* | 0.049 | 1.97 | 0.00 | 0.06 | -0.91 *(0.45)* | 0.044 | -2.02 | -1.80 | -0.03 | -0.03 *(0.02)* | -0.08 | 0.00 |
| M9: Self-Efficacy | 0.12 *(0.02)* | <0.01 | 5.81 | 0.08 | 0.16 | 0.10 *(0.36)* | 0.778 | 0.28 | -0.60 | 0.80 | 0.01 *(0.04)* | -0.07 | 0.09 |
| M10: Commitment/ Pers. Norm | 0.07 *(0.02)* | <0.01 | 3.93 | 0.04 | 0.11 | -0.36 *(0.41)* | 0.377 | -0.88 | -1.16 | 0.44 | -0.03 *(0.03)* | -0.08 | 0.02 |
| M11: Action Planning | 0.45 *(0.02)* | <0.01 | 18.70 | 0.40 | 0.50 | 3.86 *(0.32)* | <0.01 | 11.90 | 3.23 | 4.50 | *1.73 (0.17)* | 1.41 | 2.09 |
| M12: Coping Planning | 0.01 *(0.03)* | 0.869 | 0.16 | -0.06 | 0.07 | -0.54 *(0.23)* | 0.022 | -2.30 | -0.99 | -0.08 | 0.00 *(0.02)* | -0.04 | 0.03 |
|  |  |  |  |  |  |  |  |  |  |  |  |  |  |
| Control vs. CLTS+RANAS_ComPlan | Intervention (a-path) | |  |  |  | Latrine construction (b-path) | | | |  | Indirect effects (a*b-path) | | |
|  |  |  |  | CI_95_ |  |  |  |  | CI_95_ |  |  | CI_95_ |  |
|  | Estimate *(SE)* | *p* | OR | LL | UL | Estimate *(SE)* | *p* | OR | LL | UL | Estimate *(SE)* | LL | UL |
|  |  |  |  |  |  |  |  |  |  |  |  |  |  |
| M1: Vulnerability | 0.00 *(0.03)* | 0.973 | -0.03 | -0.05 | 0.05 | -0.30 *(0.19)* | 0.108 | -1.61 | -0.67 | 0.07 | 0.00 *(0.01)* | -0.02 | 0.02 |
| M2: Severity | 0.04 *(0.02)* | 0.043 | 2.03 | 0.00 | 0.08 | 0.53 *(0.26)* | 0.044 | 2.02 | 0.02 | 1.05 | 0.02 *(0.02)* | 0.00 | 0.06 |
| M3: Factual Knowledge | -0.01 *(0.01)* | 0.389 | -0.86 | -0.03 | 0.01 | 0.06 *(0.63)* | 0.929 | 0.09 | -1.17 | 1.28 | 0.00 *(0.01)* | -0.02 | 0.01 |
| M4: Affective Beliefs | -0.04 *(0.01)* | 0.007 | -2.68 | -0.07 | -0.01 | -0.34 *(0.44)* | 0.442 | -0.77 | -1.21 | 0.53 | 0.01 *(0.02)* | -0.02 | 0.06 |
| M5: Instrumental Beliefs | -0.04 *(0.02)* | 0.024 | -2.26 | -0.07 | -0.01 | 0.17 *(0.37)* | 0.649 | 0.46 | -0.55 | 0.89 | -0.01 *(0.02)* | -0.05 | 0.02 |
| M6: Descriptive Norms | 0.56 *(0.02)* | <0.01 | 29.83 | 0.53 | 0.60 | 2.77 *(0.32)* | <0.01 | 8.71 | 2.14 | 3.39 | 1.56 *(0.20)* | 1.17 | 1.93 |
| M7: Injunctive Norms | 0.27 *(0.02)* | <0.01 | 10.98 | 0.22 | 0.32 | 0.26 *(0.27)* | 0.342 | 0.95 | -0.27 | 0.79 | 0.07 *(0.07)* | -0.07 | 0.22 |
| M8: Action Knowledge | 0.00 *(0.01)* | 0.976 | 0.03 | -0.03 | 0.03 | -0.09 *(0.44)* | 0.830 | -0.21 | -0.96 | 0.77 | 0.00 *(0.01)* | -0.02 | 0.01 |
| M9: Self-Efficacy | 0.12 *(0.02)* | <0.01 | 5.97 | 0.08 | 0.16 | -0.26 *(0.35)* | 0.450 | -0.75 | -0.95 | 0.42 | -0.03 *(0.04)* | -0.11 | 0.04 |
| M10: Commitment/ Pers. Norm | 0.10 *(0.02)* | <0.01 | 5.87 | 0.07 | 0.14 | 0.07 *(0.40)* | 0.868 | 0.17 | -0.73 | 0.86 | 0.01 *(0.04)* | -0.06 | 0.08 |
| M11: Action Planning | 0.43 *(0.02)* | <0.01 | 18.58 | 0.39 | 0.48 | 3.71 *(0.33)* | <0.01 | 11.12 | 3.06 | 4.36 | 1.61 *(0.16)* | 1.30 | 1.93 |
| M12: Coping Planning | 0.06 *(0.03)* | 0.037 | 2.09 | 0.00 | 0.12 | -0.56 *(0.24)* | 0.021 | -2.31 | -1.03 | -0.08 | -0.03 *(0.02)* | -0.09 | 0.00 |

*Note.* Multiple mediation analysis (PROCESS, IBM SPSS Statistics 24), outcome variable: latrine construction, coded 0=no latrine, 1=latrine (finished or under construction). *SE*= standard error. *OR*= Odds Ratio. *CI*=confidence interval. Control: *n=* 633, CLTS_only: *n=* 593, CLTS+RANAS-Com: *n=*508, CLTS+RANAS-Plan: *n=*467, CLTS+RANAS-ComPlan: *n=*502.

Rossiter, J.R. (2011). Marketing measurement revolution: The C‐OAR‐SE method and why it must replace psychometrics. *European Journal of Marketing,* 45, 1561-1588.
